# A Low-Cost System Using a Big-Data Deep-Learning Framework for Assessing Physical Telerehabilitation: A Proof-of-Concept

**DOI:** 10.3390/healthcare11040507

**Published:** 2023-02-09

**Authors:** José Miguel Ramírez-Sanz, José Luis Garrido-Labrador, Alicia Olivares-Gil, Álvaro García-Bustillo, Álvar Arnaiz-González, José-Francisco Díez-Pastor, Maha Jahouh, Josefa González-Santos, Jerónimo J. González-Bernal, Marta Allende-Río, Florita Valiñas-Sieiro, Jose M. Trejo-Gabriel-Galan, Esther Cubo

**Affiliations:** 1 Escuela Politécnica Superior, Departamento de Ingeniería Informática, Universidad de Burgos, Avda. Cantabria s/n, 09006 Burgos, Spain; 2 Fundación Burgos por la Investigación de la Salud, 09006 Burgos, Spain; 3 Departamento de la Salud, Facultad de Ciencias de la Salud, Universidad de Burgos, Paseo Comendadores s/n, 09001 Burgos, Spain; 4 Servicio de Neurología, Hospital Universitario de Burgos, 09006 Burgos, Spain

**Keywords:** Parkinson’s disease, telerehabilitation, telemedicine, big data, artificial intelligence in healthcare

## Abstract

The consolidation of telerehabilitation for the treatment of many diseases over the last decades is a consequence of its cost-effective results and its ability to offer access to rehabilitation in remote areas. Telerehabilitation operates over a distance, so vulnerable patients are never exposed to unnecessary risks. Despite its low cost, the need for a professional to assess therapeutic exercises and proper corporal movements online should also be mentioned. The focus of this paper is on a telerehabilitation system for patients suffering from Parkinson’s disease in remote villages and other less accessible locations. A full-stack is presented using big data frameworks that facilitate communication between the patient and the occupational therapist, the recording of each session, and real-time skeleton identification using artificial intelligence techniques. Big data technologies are used to process the numerous videos that are generated during the course of treating simultaneous patients. Moreover, the skeleton of each patient can be estimated using deep neural networks for automated evaluation of corporal exercises, which is of immense help to the therapists in charge of the treatment programs.

## 1. Introduction

Parkinson’s disease (PD) is a progressive neurodegenerative condition, causing primary impairment of the motor system [1]. The prevalence of PD has been increasing as the world’s population ages, even surpassing the growth of Alzheimer’s disease. According to the Global Burden of Disease study, the number of people living with PD doubled to 6.2 million patients between 1990 and 2015, an increase that is expected to increase twofold by 2040 [2]. PD is a chronic and debilitating disease, especially among older people, and it imposes high financial burdens on patients, families, and healthcare systems [3,4].

PD is characterized by motor symptoms, including bradykinesia (slowness), rigidity, and resting tremor caused by moderate to severe dopaminergic neuronal losses in the ’substantia nigra pars compacta’ area [5,6]. Although PD is still considered a paradigmatic movement disorder, it is accompanied by non-motor symptoms unrelated to motor dysfunction, such as cognitive impairment, behavioral disturbances, hyposmia, sleep disorders, and autonomic dysfunction, even from the onset of the first pathological symptoms [7]. Non-motor symptoms may become dominant in clinical history as the disease progresses, with significant implications for quality of life and caregiver burdens [8]. Among the non-motor symptoms, cognitive impairment is characterized by visuospatial deficits and dysfunctional executive processes that impede goal-directed activities [9]. In consequence, even non-demented patients with mild to moderate PD may encounter difficulties with planning, organizational skills, and concentration while performing daily activities and walking [10].

Studies have revealed substantial reductions in physical activity among people with PD compared with healthy older adults, even at the earliest stage of the disease [11]. As PD progresses and as a consequence of decreased physical activity, falls that are often harmful among patients living with PD are very common [12]. Approximately 40–70% of people with PD fall each year, and one-third fall repeatedly [5,12]. Overall, people with PD are twice as likely as the healthy elderly population to suffer a fall [13]. There is evidence to show that falls have major impacts on the lives of people with PD, including debilitating effects on confidence [14], decreased activity levels, and reduced quality of life [15,16]. Moreover, falls are financially costly for individuals and healthcare systems. Repeated falls are risk factors that imply subsequent falls, often with such consequences as fractures and prolonged immobility before help arrives. In contrast, the fear of falling can lead to dependency and social isolation [17]. Although the incidence of falls increases with disease severity, falls are even common in the early stages of the condition [18].

Anti-Parkinsonian drugs are the main treatments used to counter the symptoms that can cause PD patients to fall, while research into finding a cure continues. However, medication is not sufficient to relieve reduced postural control and prevent falls, especially among patients with advanced PD [5]. Other fall-prevention strategies include educational support, physical exercise, and modification of environmental factors. In this regard, behavioral change strategies provide a theoretically based approach for helping patients with PD to develop the skills they need (e.g., goal setting and self-monitoring) for successful engagement in sustained exercising [19], and, therefore, fall prevention. Several meta-analyses published in the literature have concluded that exercise as a single intervention can prevent falls among community-dwelling patients with PD and can be particularly beneficial for maintaining postural control, mobility, and daily living activities [20]. In addition, there is growing evidence that remote monitoring of physical activity is also feasible in neurological diseases, including PD [19].

In Spain, PD associations often offer long-term physical therapy activities for patients. According to the PD Federation of Spain, the population of people living with PD was estimated at approximately 92,971 (73,044 to 116,691) in 2016 [2], of whom 12,800 patients (13.7%) had access to physical therapy and even lower ratios are likely in rural areas.

In this scenario, the role of information and communication technologies (ICT) in a home fall prevention program for patients living with PD could be crucial. In fact, a multidisciplinary home fall prevention program running on an ICT platform represents a promising, cost-effective strategy [21,22], with the added advantage of reaching PD patients remotely. This need can be a priority considering the devastating effects of the COVID-19 pandemic on vulnerable populations with chronic diseases, such as PD. During this time of crisis, many patients with PD are likely to benefit from restored access to general care services via telemedicine [23,24].

Different software solutions have been described in previous literature studies on telerehabilitation, such as the Platform of Telemedicine (Teleriab), which is designed to offer telerehabilitation services. Health staff can contact patients through this web platform either over the phone or by using a video-conferencing option with one or more patients either simultaneously (with a maximum of eight simultaneous video conferences) or consecutively respecting the privacy of the patients [21]. Other authors have provided telerehabilitation through video-conferencing via a smartphone or iPad [25,26] and with the assistance of in-home three-dimensional biometrics and a (digitally simulated) avatar coach [27]. Both the systems and the tools that are designed to facilitate home-based telerehabilitation software systems have increased over the past decades and we recommend the work of Hosseiniravandi et al. for an up-to-date review of these systems [28].

However, although the feasibility of telerehabilitation has been shown in the previous literature studies, there are still few comprehensive studies aimed to incorporate big data technology and artificial intelligence to facilitate the work of PD physical therapists. In this paper, a low-cost telemedicine home telerehabilitation treatment system for PD patients is presented. The system uses big data technologies and artificial intelligence for helping therapists to identify whether the patients are performing their exercises properly. The system is currently used in a randomized controlled trial in the framework of a national research project of the Spanish Government for improving the quality of life of PD patients living in the community. In summary, the main goals of this paper are:

- To present the development of a low-cost system for physical telerehabilitation.
- The use of big data and artificial intelligence technologies to identify the patients’ skeleton while performing telerehabilitation in real time.
- The evaluation of the system in a randomized controlled trial for PD patients’ fall prevention.

Moreover, this paper tries to answer the following research questions:

- RQ1: Is it possible to enable telerehabilitation for PD patients using low-cost systems?
- RQ2: Is it possible, with these low-cost systems, to process telerehabilitation exercises in real time?
- RQ3: Is the system easy to install and use?

The rest of this paper is organized as follows. In Section 2, the related works are presented. In Section 3, the materials and methods used for telerehabilitation will be reviewed and the software telerehabilitation system that is a central proposal in this paper will be presented. In Section 4, the main impact of this work and its limitations are discussed and, finally, in Section 5 the future research lines are presented.

## 2. Related Works

Hereinafter, the related works with telerehabilitation will be presented, especially the techniques for unsupervised telerehabilitation.

PD patients suffer from many distance-related barriers, a disability that worsens over time, and irregular access to specialized health professionals, all of which are problems that limit their access to care programs [29]. Nonetheless, the use of telecommunication technologies offer these patients new possibilities both for telerehabilitation programs and follow-up visits for patients in remote areas who may be unable to travel [30,31].

The application of telerehabilitation is an expanding option in various health scenarios. Nevertheless, there are some limitations that must be taken into account [32]. In particular, the current COVID-19 pandemic has revealed the need and opportunity for telemedicine in future healthcare systems. The main concern is to ensure that the patient is performing the therapeutic movements properly and safely throughout the exercises [33].

Movement analysis is crucial for identifying and evaluating neuromuscular problems. It is also appropriate for monitoring the development of pathologies such as PD, because the evaluation is mainly visual and it can be performed using systems cataloged in one of the following 4 categories: (1) highly accurate 3D specialized systems, (2) movable capture systems, (3) wearable wireless sensors, or (4) video-game handheld controllers.

- It is believed that 3D specialized systems yield the best results [34]. These systems consist of numerous high-resolution cameras that are operated in controlled environments. The main disadvantages of these systems are their costs (several thousands of dollars) and the need for a controlled environment operated by specialized personnel. Some examples are Vicon (Vicon Motion Systems, Oxford, UK) [35], Optotrak (Northern Digital, Inc., Waterloo, ON, Canada), and Prime (NaturalPoint, Inc., Corvallis, OR, USA).
- Mobile capture systems (Microsoft Kinect is one of the best-known) can be installed in uncontrolled and mainly indoor environments. These systems are capable of full-body motion capture in 3D. Kinect has been used as a platform for rehabilitation exercises [36]. Less expensive standard 2D monocular cameras may also be included within this category; they are now also capable of tracking on-screen postures thanks to the appearance of modern deep-learning algorithms (e.g., [37]). Systems based on single 2D cameras in combination with deep learning algorithms have recently been used in the field of rehabilitation. In [38], they were used for the analysis of shoulder rehabilitation exercises and in [39] a client-server system, and the Deep Learning OpenPose framework was used to identify movements of any part of the body. This last work used a smartphone as the only device in the patient’s home, being the system able to estimate the position of the body in exercises previously recorded with this smartphone. Nonetheless, it was not capable of making the estimation in real time.
- Wearable sensor technology is portable and can capture data in uncontrolled environments, even outdoors. These systems are also much less expensive than the specialized 3D capture systems. Wearable sensors have previously been used to monitor patients with PD [40]. However, these same systems are very sensitive to correct placement and calibration, which makes their use very difficult, especially among older patients who have no assistance at that time.
- Video-game handheld controllers. The popular Nintendo Wii console has been used for the treatment of PD patients [41] and stroke rehabilitation [42]; Oculus Quest 2 has also previously been used in shoulder rehabilitation [43]. These systems can perform arm positioning quite precisely, using a combination of accelerometers and optical sensor technology. They are easy to use, but their main limitation is that they only are able to evaluate arm exercises.

To the best of our knowledge, no system has been created that meets all the following requirements, i.e., inexpensive, easy to install and use, capable of working in real time, and able to analyze all kinds of movements that may involve joints throughout the body. Systems based on movement capture using single 2D cameras and deep learning techniques are the closest to solving these limitations.

Table 1 shows a summary of the main characteristics of the movement analysis systems described above.

### 2.1. Monocular Human Pose Estimation

Monocular human pose estimation (MHPE) is a technique that captures the posture of the human body from monocular images and videos. MHPE is more challenging than other approaches that use specialized hardware, such as binocular cameras and depth sensors. These challenges are related to the complexity of the human body shape and the high degree of freedom of the extremities, which can produce self-occlusions. They are also related to the high variability of physical profiles that are accentuated by the great variety of clothes, backgrounds, and viewing angles [44]. There are a large number of algorithms depending on the type of input images and the type of output (skeleton, contour, and volume) to be estimated.

Focusing on obtaining 2D skeletons from single images, one of the most relevant methods for this task is Mask R-CNN [45]. Mask R-CNN addresses the problem of segmentation of instances, consisting of detecting and delineating each object of interest in the image. This problem is a combination of object detection (finding and classifying objects in an image) and semantic segmentation (defining the image at the pixel level that outlines the object).

Mask R-CNN is a neural network architecture, which achieves state-of-the-art results in image segmentation. It is an extension of the Faster R-CNN detector [46,47]. While the previous architecture only generates and classifies bounding boxes containing objects of interest, Mask R-CNN also generates the segmentation mask, using a fully convolutional network (FCN) model for semantic segmentation [48]. Mask R-CNN was adapted to estimate poses in order to detect and classify each key articulation of the skeleton separately (e.g., left shoulder, right elbow...). Mask R-CNN is, according to its authors, capable of achieving state-of-the-art results in COCO 2016 keypoint detection while running at five frames per second (fps). As open-source software, it is available through the Detectron2 library (https://github.com/facebookresearch/detectron2, accessed on 5 of March 2020).

### 2.2. Automatic Evaluation of Motions

The automatic evaluation of movements can, objectively, determine when a movement is properly performed. These types of systems have been used to analyze sporting performance [49], to supervise the training of medical personnel [50], and for rehabilitation.

The methods developed in this field can be classified into two categories:

- Rule-based methods. These systems require a set of rules to be defined by experts. These rules must capture the key features of each movement that should be executed and evaluated. There are multiple examples in both, sports evaluation [51] and telerehabilitation [52]. This approach requires little computational power and can provide feedback with specific information for experts, such as certain joints not being flexed at a certain angle. As disadvantages, this approach is more prone to generating false positives (incorrect execution of movements) due to errors in the capture sensors; and the set of rules for each move is required to be carefully defined by experts, which can be very difficult in complex moves (can only be applied to specific gestures with quantifiable characteristics). This is an approach with little flexibility or adaptability since modifying an exercise requires a complete review of the set of rules associated with it. An attempt has been made to solve this negative aspect by expressing the rules using a reusable and customized encoding based on eXtensible Markup Language (XML) [53].
- Template-based methods. In this approach, a sequence of movements is recorded a priori, and later, it is used to analyze other sequences of the same movement, carried out by other people. There are two subcategories:
  - Distance-based methods. The sequence of movements previously recorded is compared with the observed movements. The system obtains a distance between the movement made by the expert and the movement made by the trainee (e.g., the patient undergoing rehabilitation). A movement can be characterized as the position of the skeletal articulation at each point in time. Usually, the movements are aligned using techniques such as dynamic time warping (DTW) [54] to deal with different execution speeds. Normalization techniques are also used to decrease the influence of the morphology of each individual [55]. This approach is used, for example, in [56], where DTW is used to compare how patients performed a movement before, during, and after treatment.
  - Model-based methods. In this case, the recorded sequence of movements is used to train a model that can later be used to classify the observed movement. For example, in [57], a model training-based approach is used to classify movements between “good” and “poor” mobility in patients suffering from age-related impairments. From each frame of a video, a 3D skeleton is extracted using the Kinect camera. This skeleton is processed to obtain a fixed-size instance with numerical attributes, such as the center of mass (CoM), Euclidean distance between the shoulders and wrist, Euler angle between the torso and legs, etc. A motion, therefore, is a length-dependent size list of frame instances. Cluster analysis is used to determine the centroids of each movement, which are subsequently concatenated to create an instance that represents the entire movement. Finally, a classification algorithm, such as random forest or SVM, is trained with a dataset made up of movement instances collected from all patients.

Moreover, some authors have developed hybrid systems with the best of the two categories. In [58], a HSMM (hidden semi-Markov model) is used to create a solution that can provide reliable quantitative feedback to physiotherapists while being able to generalize to different types of exercises.

## 3. Materials and Methods

The use of web-based video conference visits has proven feasible for the treatment of PD, offering similar results to in-office visits [59]. The two main cornerstones of a successful telerehabilitation system are an affordable device/technology and ensuring the patient is doing the exercises properly [60]. Obviously, the final aim of telerehabilitation exercises is to improve clinical outcomes and the patient’s quality of life.

The exercises prescribed by physical therapists for several diseases are commonly unsupervised, i.e., the patient exercises at home without the supervision of a therapist. It usually occurs because the logistics of having a therapist supervising the patients online are usually too expensive [61]. One drawback of unsupervised exercises is their lower efficacy and safety; for instance, falls during their execution can be cumbersome.

In this section, the target population of this study is presented, the physical exercises of the therapist are explained, and the technology behind the telerehabilitation is presented.

### 3.1. Target Population

A total of 76 patients were included in the study, of which, 38 patients formed the intervention group and received telerehabilitation in addition to standard medical treatment. The other 38 patients, who formed the control group, received only standard medical treatment. The patients were invited to participate in the study based on the following criteria: they were 18 years old or more, had been diagnosed with idiopathic PD using the MDS criteria [62,63], have a moderate-high risk of falling (determined by a history of at least one fall in the previous 12 months) and a Hoehn and Yahr stage of less than 3 [64]. They also had to have a Montreal Cognitive Assessment score of over 18 [65] and not have access to non-pharmacological therapies at that time. Furthermore, they had to meet one of the following additional criteria: the presence of freezing of gait or self-selected gait speed of less than 1.1 m per second [66].

Patients with severe psychiatric or cerebrovascular diseases, severe traumatic head injuries, severe orthopedic lower limb or spine problems, peripheral neuropathy, rheumatological disease, other systemic diseases, or sensory deficiencies that, according to the specialist, might interfere with the study, were not considered for the study, as telerehabilitation was not advisable for them.

### 3.2. Rehabilitation

The telerehabilitation program is individualized for each patient. All sessions are directed and supervised in real time by the occupational therapist. During telerehabilitation sessions, the occupational therapist provides the necessary instructions for the patient to perform the various exercises.

The therapist and the patient begin each session with a physical warm-up, which consists of a series of mobility exercises and stretching. The therapist instructs the patient to mobilize muscles and articulations, beginning with neck movements and ending with foot movements. On-screen visualization means that the therapist is able to show the patient the correct way of performing each movement and then instruct the patient to imitate it.

**Figure 1:**
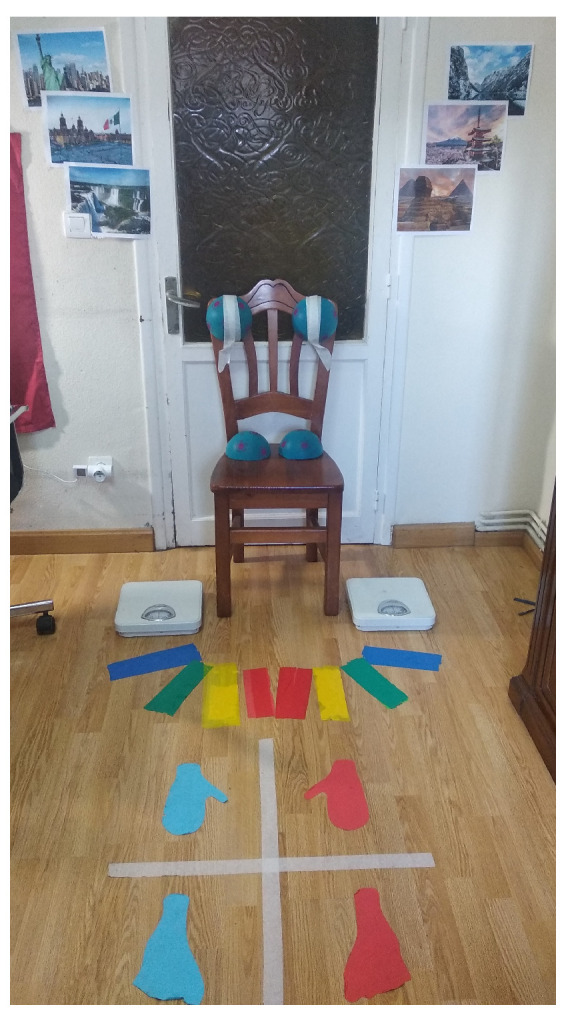
The setup of a patient’s room with the equipment on the wall and the floor for the rehabilitation exercises.

The rest of the exercises are designed to improve body posture, increase mobility, promote movements, reduce axial stiffness, decrease dyskinesia and gait freezes, and improve balance and coordination. Figure 1 shows the setup of a patient’s room where the equipment on the wall and on the floor are used in the following exercises:

- First: rotations of the body trunk in a sitting position. In this exercise, the patient is asked to hold a ball with both hands and rotate the trunk to both sides of the body, following the instructions of the therapist. Then, the patient is asked to move the ball toward a series of images placed on the wall behind the patient.
- Second: mobilizations of the lower extremities. In this exercise, the patient is in a sitting position and they must move both feet following the therapist’s instructions, placing them on a series of colored cards arranged on the floor.
- Third: weight exercises with the lower extremities. In these exercises, the patient is in a sitting position and places both feet on a weighing scale, while trying to move the needle on the scales to the level that the therapist might indicate.
- Fourth: straightening reactions. Going from sitting to standing, and vice versa, the patient is asked to place the body in various postures, on a continuum from sitting to standing up.
- Fifth: mobility, balance, and coordination exercises. In a standing position, the patient is asked to follow the therapist’s instructions, moving and placing both feet in each of the four quadrants on the floor.

### 3.3. Technology

**Figure 2:**
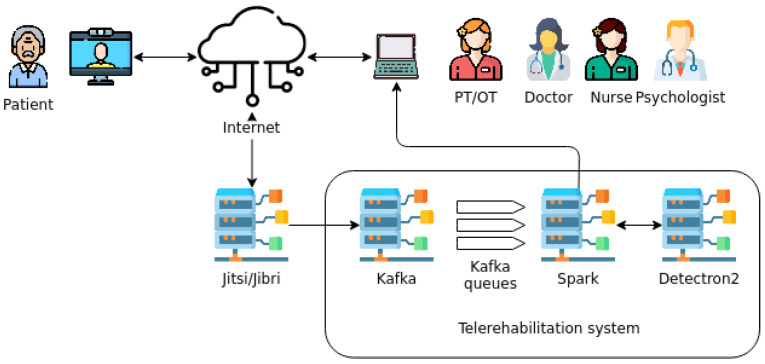
Schematic view of the different components of the telerehabilitation system.

The telerehabilitation system presented in this study is composed of the (*Jitsi*) server through which the data is relayed, an affordable device that patients can use at home (in combination with a television), and an additional server for applying the Artificial Intelligence (AI) techniques (with Kafka, Spark, and Detectron2). The user interface is responsive, i.e., the therapists and the doctors can access the system through their personal computers or portable devices, such as smartphones or tablets. The big data architecture used in this paper ensures maximum data transfer rates for immediate communication and ease of use. A schematic view of the telerehabilitation system and the actors involved can be seen in Figure 2.

#### 3.3.1. Device and Servers

For this study, a first attempt was made using a Raspberry Pi 3. This device was selected because of its low cost and user-friendliness, but the device was not able to make the call and record the video at the same time due to overheating. To solve this problem a new device needs to be chosen. We selected the MSI—Cubi N 8GL-001BEU N40000 PC equipped with an Intel^®^ Celeron^®^ N4000 1.10 GHz processor, with 4 GB of RAM and a 120 GB capacity SSD. The PC also had a Logitech HD Pro WebCam C920 with an integrated microphone. Both devices (PC and camera) were installed in each patient’s home.

**Figure 3:**
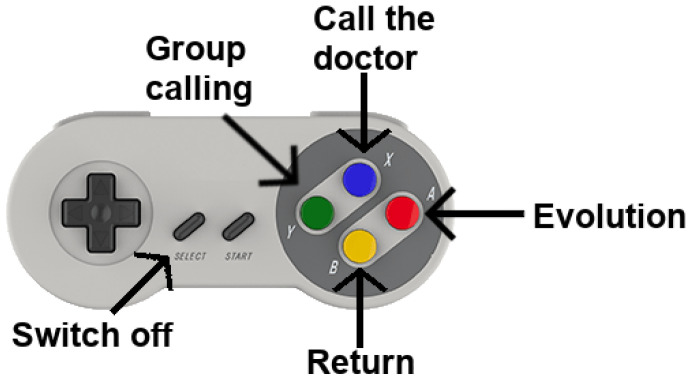
SNES controller pad to operate the application showing the function associated with each button.

**Figure 4:**
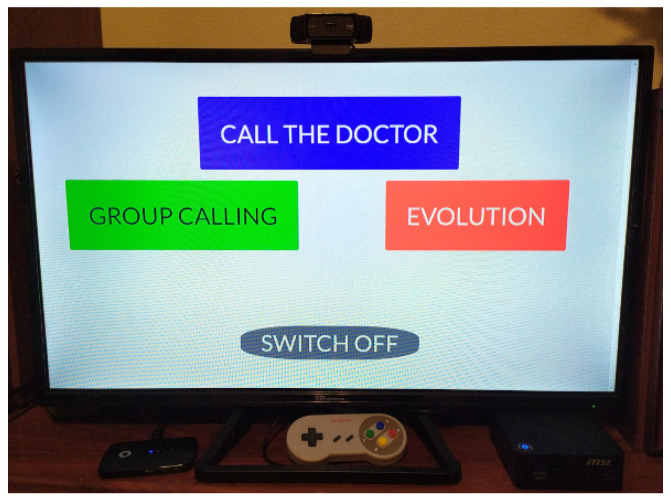
Installation of the equipment at the patient’s house with its main interface on the screen.

A Super Nintendo Entertainment System (SNES) controller pad (Figure 3) is used to control the device, in which each colored button controls a specific action that is shown on the screen with the same color (Figure 4). The available actions are as follows: a call to the occupational therapist/medic (blue button), group call with the psychologist (green button), query evolution (red button), and go back (yellow button). The benefits of this pad are: its low cost in comparison with other alternatives, its large buttons, and its easy-to-remember colors.

The call system consists of an open source video-bridge Jitsi deployed on a Google Cloud Instance with one virtual CPU and 3.75 GB of memory, under the name *n1-standard-1*. The use of a private *Jitsi* instance improves security, as its availability is limited to the authorized users, i.e., patients and medical staff involved with the study.

An *Intel^®^ Xeon^®^ E5-2630 v4* 10-core server, with 3 GPUs, Nvidia Titan Xp, and 128 GB of memory is used for the video processing and the application of the AI techniques.

#### 3.3.2. Big Data Architecture

The design of the architecture was based on the ETL (extract, transform, and load) process [67]. Firstly, the video frames are extracted from the communication between the occupational therapist and the patient. These data are transformed by computer visual analytics to obtain relevant information and, finally, the results are relayed to the occupational therapist’s screen and stored on persistent data media.

The information must be extracted, transformed, and loaded as fast as it is transmitted, to generate an output stream with at least the same input speed, so that the information can be displayed in real time to the occupational therapist. Thus, each data item has to be processed consuming as little memory and CPU time as possible and ensuring no frames loss as they were relayed.

As video-stream processing can be complex and slow, it is necessary to process it in parallel so that it guarantees an output with the same frame rate, even though a slight delay is generated between the patient’s video and the information that is relayed from it.

The frames are received through a UDP (user datagram protocol) stream that sends Jibri (JItsi BRoadcaster Infrastructure) (available on https://github.com/jitsi/jibri, accessed on 1 of October 2017) to the processing server that transfers the data into an *Apache Kafka* platform. *Apache Kafka* serializes the frames in *JPEG/Exif* at 95% with *ZLib* compression, guaranteeing a very small size within a short time, which facilitates ’enqueue’ and ’dequeue’ speeds on the *Kafka* platform.

The frames are processed and transformed by an *Apache Spark Streaming* application that parallelizes the process in various workers for image processing, as will be explained later. Finally, the processed frames can be projected on the occupational therapist’s screen, allowing them to enhance the therapy.

The selection of the number of workers is crucial, to ensure the proper processing speed. To do so, it is necessary to know the average time ($t_{mean}$) and the standard deviation ($t_{std}$) of processing one frame. Using these two values, Equation (1) returns the minimum number of workers needed for keeping the same frame rate as output.

(1) $$\left\lceil \frac{(t_{mean}+2t_{std})*\mathrm{fps}}{1000}\right\rceil$$

The values of $t_{mean}$ and $t_{std}$ depend on the hardware on which the application is running. Moreover, there is a limit on the number of workers that can be run on one machine, because they have to share certain resources.

**Figure 5:**
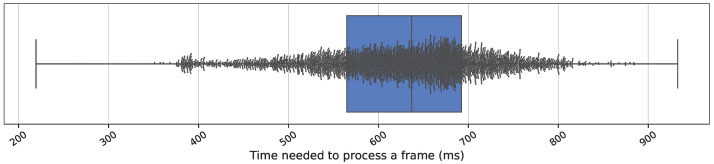
Boxplot of processing time for each frame. A total of 4863 frames from a set of 16 videos were processed to obtain values and to determine the number of workers.

As previously noted, in this study an *Intel^®^ Xeon^®^ E5-2630 v4* processor and *NVIDIA GeForce^®^ Titan Xp* GPUs are used, with $t_{mean}$ and $t_{std}$ values of 626 ms and 98.7 ms, respectively. These values were obtained by processing a total of 16 videos, yielding the results shown in Figure 5. Therefore, 9 workers are needed when processing a video of 10 fps.

#### 3.3.3. Video Processing

The input video must be processed in real-time to identify the skeleton of the patient (making it possible to determine whether the exercises are properly performed). In order to use these images in research and in the development of the application they must be anonymized. This is done by blurring the faces in each frame to ensure that a patient can not be identified in the video recording. Notice that this step can be skipped if the application is deployed in production since the videos would be processed automatically and the only people able to see the patient’s face would be their therapist. In this case, the number of real-time calculations and, therefore, the total computational cost would be reduced.

**Figure 6:**
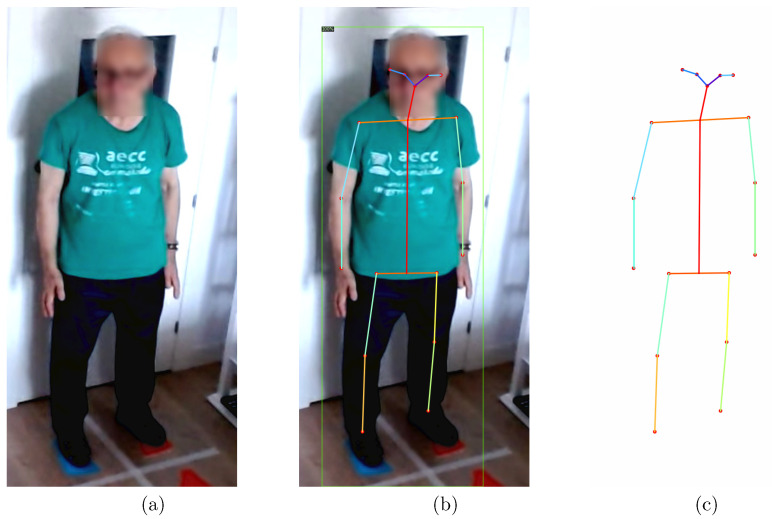
An example frame of a standing-up patient starting the exercises and the output of *Detectron2*: anonymized (**a**), anonymized with the skeleton overlapped (**b**), and the points that represent the skeleton on a white background (**c**).

A *Caffe* [68] model is used to detect the area around the face to be blurred, specifically the res10_300x300_ssd_iter_140000 for *OpenCV*. The use of a 3×3 Gaussian filter guarantees anonymization, while ensuring that the following processing steps are correctly performed. Figure 6a shows the result of a single frame after the anonymization process.

A study was performed on different video feature extraction tools as the *Facebook AI* tool called *Detectron2* and *Google’s PoseNet* tool. The objective was to choose the best *Python* computer vision tool capable of returning the main points of a human body posture. After this study, *Detectron2* was chosen.

*Detectron2* is a tool to work with different computer vision problems [69]. It provides multiple models created with the *PyTorch* [70] machine learning library. All these models incorporate deep neural networks and were trained with the *Common Objects in Context* (COCO) image dataset [71]. In this study, *COCO Person Keypoint Detection Baselines with Keypoint R-CNN* models were selected, which can identify the main articulations of a human body.

A further study was performed to select the best model for this type of problem, between the four models that *Detectron2* has available for this sort of task. An experimental test was designed to select the model with the best trade-off for this study. In the experiment, all models were run with 7 different videos (1989 frames in total) to compare the different performance times for both processing and loading (the model was reloaded for each video). The results are shown in Table 2 where the lowest value for each column is highlighted in boldface. In view of these results, keypoint_rcnn_R_50_FPN_3x was the best model for this problem, as it had the best loading time (for a considerable difference from the rest) and almost the same processing time as the fastest one. This model is a feature pyramid network (FPN) [72] with a residual network (ResNet) [73] and a region-based convolutional neural network (RCNN) [74].

Having selected the model, the main parameter that has to be configured is the threshold value of the model. The threshold expresses the sensitivity of the model to decide whether a person is or is not within the image. The higher the threshold, the greater the confidence required by the model to classify as a person. In this problem there is only one person who is fairly centeral in the image, so a high value for the threshold (0.99) is taken to ensure that the model only detected the patient (i.e., avoiding false positives in person identification).

For each frame, the output of the model consists of a series of tensors. In the main tensor, the 17 two-dimensional points represent the positions of the main articulations of a human body, such as wrists, elbows, shoulders, knees, and ankles. An example of the skeleton detected by the model in a frame with a patient can be seen in Figure 6b,c.

Some other features are computed from these points. These additional features are middle points, for example, the point of the base of the neck or the hip. Finally, 12 angles are calculated to represent the posture of the articulations and to obtain a measure that is not directly dependent on a patient’s position in relation to the camera. A disadvantage of 2D images is shown here, as these angles only remain invariant when the movement occurs in the 2D image plain. A possible option would be using 3D images, which would require more expensive devices that are incompatible with this project.

Once the posture is represented within the angles, comparisons between the postures are straightforward, just by calculating the difference between these angles.

**Figure 7:**
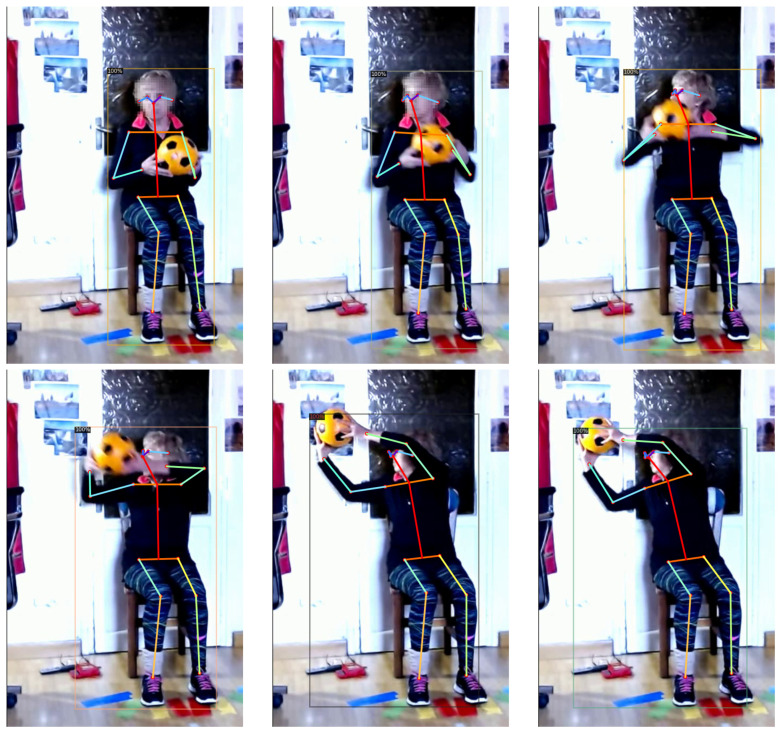
A sequence of six frames of a patient doing an exercise. The frames are anonymized and the skeleton detected by *Detectron2* is overlapped on each frame.

The patients’ skeleton data can be used by the therapist to check whether the exercises are properly performed (see example in Figure 7). Moreover, this information could be used by the system to detect the improvements of the patients over time, comparing the evolution of these angles throughout the treatment.

## 4. Discussion

Physical exercises have demonstrated their usefulness for fall prevention among PD patients. Telerehabilitation has likewise emerged as a convenient opportunity for remote monitoring of patients while they perform these exercises. Nevertheless, online rehabilitation requires a therapist for each patient during the session (as with traditional rehabilitation), which increases the cost of the therapy.

Regarding RQ1: Is it possible to enable telerehabilitation for PD patients in low-cost systems? Unlike other expensive alternatives of movement capturing (such as those that need several cameras or intrusive motion detection systems, see Table 1), the solution presented in this paper is able to identify the patients’ skeletons only by using a simple webcam and AI vision techniques. The equipment that was installed in each patient’s home has a value of around 220€, which indicates its low-budget compared with other alternatives.

Regarding RQ2: Is it possible, with these low-cost systems, to process telerehabilitation exercises in real time? The proof-of-concept of the software architecture presented here demonstrates that a low-cost telerehabilitation system running AI algorithms within big data frameworks can be of assistance to the therapist when analyzing the degree to which the exercises are properly performed by the patient. Moreover, the infrastructure was designed to manage multiple telerehabilitation sessions simultaneously using big data frameworks (*Apache Kafka* and *Apache Spark*). For each telerehabilitation session, *Kafka* receives 175.53 megabytes of video per second that must be processed in real-time. In 1 h, the server stores an average of 10.53 Gigabytes of video (i.e., 36,000 frames). Therefore, considering that, in each frame, 12 angles are identified, the dataset correspondent to a 1-hour session has 12 features and 36,000 instances of angle positions: a total of 432,000 angles.

This paper can be seen as a step forward toward weakly supervised (or unsupervised) telerehabilitation with low-cost equipment, so a therapist could be in charge of patient rehabilitation in real time.

The administration of a program supported by new home-based technologies may contribute to a new approach to prevent home fall risks among this vulnerable population and to facilitate their treatment. We aimed to make the program feasible in terms of patient and provider compliance, which represent possible barriers to the successful implementation of fall-prevention strategies [75].

Regarding RQ3: Is the system easy to install and use? The system has been successfully installed in 38 homes with very different layouts and, some of them, with quite limited space. The devices used are small and there is no need for the webcam to be exactly centered, as long as it is somewhere near the screen and it is able to capture the setup in the frame. As the devices were almost fully configured in advance, the installation and the patient’s training were completed, on average, in one hour. The system has also been designed for easy use (so that users are less likely to abandon it due to its difficulty). To assess this, the patients were asked to answer the Telehealth Usability Questionnaire (TUQ) [76] at the end of the study. The TUQ consists of 21 questions with 7 possible answers (1—strongly disagree, 7—strongly agree) and was developed to evaluate the usability of telehealth implementation and services. The final score is calculated as the sum of the scores of each question (being 147 of the maximum score). To date, 15 subjects have answered the questionnaire, with a mean score of 139.13 and a standard deviation of 4.34. Moreover, the questions especially related to ease of use (i.e., 4, 5, 6, 9, 16, and 17) have a mean score of at least 6.47 and a maximum standard deviation of 1.06. These results prove that the overall experience of the subjects was very positive and they found the system easy to use.

In addition, the health staff can assign a series of scores to each session based on the quality of the patient’s physical therapy and can provide feedback and encouragement to increase motivation and adherence to the program.

Additional advantages of this system are the possibility of group video conferences between neurologists, psychologists, and nurses where multidisciplinary approaches may be needed.

Nonetheless, the software has several limitations. At a technical level, there is an important drawback in relation to the online processing of telerehabilitation. On the one hand, an important investment in hardware is necessary, particularly in graphics processing units (GPUs), which support a large number of *Spark workers* and, thus, ensure a smooth response for health staff. On the other hand, the *Jitsi* platform used for communication must be properly maintained, since we observed during the telerehabilitation that the machine’s resources were at times not properly released. Furthermore, the dependency on an internet connection in remote areas can be a problem for the smooth functioning of video conferences. The significance of this problem will lessen as connectivity improves in rural areas, due to new technologies, such as 5G and satellite technology. Currently, the telerehabilitation sector is still very immature, with a large number of devices and heterogeneous methodologies coexisting, which limits any extrapolation of the results. Nevertheless, machine learning-based systems often improve with larger amounts of data, and the creation of shared databases, despite the use of different devices, is an important issue. Health controls and standardized protocols could also be incorporated so that the results could be compared between studies using different devices.

The adoption of telemedicine services has grown exponentially over the past 5 years, and the market is expected to reach more than USD 130 billion by 2025 [77]. However, new technologies need to be integrated into these systems for them to improve, and user feedback is essential [78], as first-generation software and hardware components are never as user-friendly or efficient as subsequent generations that have been improved on the basis of that feedback. Patient and caregiver engagement is at least modest for wearable and mobile technologies and, as some authors demonstrated, 50% stopped using wearables after over a year. Similarly, there is a high abandonment rate among smartphone app users, as 74% of apps are not used more than 10 times. Lack of motivation to use new technology, including telerehabilitation, should not be underestimated, particularly in the absence of meaningful feedback provided to their users. Preliminary evidence has led to the suggestion that patient empowerment and the inclusion of patients as active players in the development of research activities may favorably impact compliance. Quality of care and adherence to telerehabilitation treatment programs using synchronous videoconferencing also deserves attention [79,80].

## 5. Future Research Lines

One of the main future lines of research is the automatic evaluation of the exercises by the patients, following other lines of work, such as [37,81]. Once the skeleton has been identified, the comparisons can be drawn along two lines: either comparing treatment sessions with the patient (for assessing improvements over the course of time) or comparing the exercises that the therapist proposes (evaluating the differences between the gold standard and patients’ exercises). For this purpose, DTW could be used to obtain where an exercise starts and where it ends. Then, the evaluation method will be applied to obtain a nominal or numerical result of the patients’ performance.

A possible future line is to replace a 2D pose estimation with a 3D pose estimation. Adequately estimating the Z-axis position of hands, limbs, and head can increase the quality of exercise evaluation and the number of exercises that can be evaluated. The 3D pose estimation using multiple cameras is currently the state-of-the-art approach [82,83]. However, recent algorithms have been proposed to estimate the 3D pose estimation from a sequence of 2D key points. MixSTE [84], is a novel transformer-based *seq2seq* approach, capable of producing a sequence of 3D key points from video obtained by a single camera. Using this type of algorithm in future works would not increase the cost of the solution and could lead to a much more accurate estimation of the movements.

As previously noted, one of the main concerns of unsupervised rehabilitation is the possibility of falls during the performance of the exercises. There is no doubt that a low-cost multidisciplinary telemedicine approach for home fall prevention among people living with PD is likely to become increasingly important in healthcare. With this in mind, a module of fall identification would be incorporated into the presented system that could automatically call an emergency in case of a fall is detected.

Despite challenges, the continuous improvements in technological sophistication have created opportunities to administer multidisciplinary care in PD. From a clinical point of view, telemedicine using video conferences can be used as a tool for clinical, quantitative research on fall prevention programs, such as increasing participation in clinical trials, administering e-rating scales, and measuring different PD signs, such as movements, speech, etc. On the other hand, video conferences can also be useful for qualitative research, for instance, focus group interviews, providing meaningful perspectives from patients, caregivers, and health professionals. Furthermore, the results of the economic evaluation could provide information on the cost-effectiveness of the intervention and the effects on the quality of life of patients with PD. The cost-effectiveness of the device and the treatment program could be tested and implemented in health service settings and extrapolated to other chronic and neurodegenerative diseases.

## Figures and Tables

**Table 1:** Summary table: pros and cons of movement capture systems. Background cell color highlights the suitability of the systems according to the feature, being green the best and red the worst.

	3D Specialized Systems	Movable Capture Systems (3D Cameras)	Movable Capture Systems (2D Cameras)	Wearable Wireless Sensors	Handheld Game Controllers
Sample hardware	Vicon, Optotrak, Prime	Microsoft Kinect	Logitech HD Pro C920	LilyPad, Steval	Nintendo Wii
Environment	Indoor, controlled environment	Indoor	Mainly indoor	Indoor, outdoor	Indoor, outdoor
Installation	Complex	Easy	Easy	Medium	Easy
Use	Complex	Easy	Easy	Complex	Easy
Scope	Full body	Full body	Full body	Full body	Arms
Quality	High	Medium	Medium	Medium	Medium
Price	High	Medium	Low	Medium	Medium

**Table 2:** Time comparison for *COCO person keypoint detection baselines with keypoint R-CNN* models, in milliseconds. The fastest result in each column is highlighted in boldface.

Model	Loading (ms)	Processing (ms)
keypoint_rcnn_R_50_FPN_1x	8083.19	**216,519.97**
keypoint_rcnn_R_50_FPN_3x	**7539.65**	216,625.36
keypoint_rcnn_R_101_FPN_3x	9806.49	259,716.33
keypoint_rcnn_X_101_32x8d_FPN_3x	14,853.03	407,086.70

## Data Availability

All of the source codes of the telerehabilitation system are publicly available on Github: https://github.com/admirable-ubu/FIS-FBIS.
